# Expression of pluripotency markers in the bovine uterus with adenomyosis

**DOI:** 10.1186/s12958-015-0106-0

**Published:** 2015-09-29

**Authors:** Martyna Łupicka, Barbara Socha, Agata Szczepańska, Anna Korzekwa

**Affiliations:** Department of Reproductive Immunology and Pathology, Institute of Animal Reproduction and Food Research, Polish Academy of Sciences, 10-748 Olsztyn, Poland

**Keywords:** Uterus, Pluripotent cells, Adenomyosis, Cow

## Abstract

**Background:**

Adenomyosis is a proliferative uterine dysfunction with unknown aetiology. One possible mechanism of its development involves disturbances in stem cell differentiation in uterine tissue. Previously, we identified pluripotent/multipotent cells in the bovine uterus, therefore our present study focused on determining expression of pluripotency markers, NANOG, OCT4 and SOX2, in bovine adenomyotic tissues and cells.

**Findings:**

Immunolocalisation revealed protein expression of NANOG, OCT4 and SOX2 in both normal and adenomyotic uteri. mRNA expression for *NANOG* and *OCT4* was increased in tissues obtained from uteri with adenomyosis compared to controls, but at the protein level there were no significant differences. mRNA expression for all three pluripotency markers was higher in myometrial cells isolated from uteri with adenomyotic lesions than in those isolated from normal uteri. The protein level of NANOG and SOX2 was decreased in stromal cells from adenomyotic tissues, whereas the level of OCT4 and SOX2 was increased in myometrial cells obtained from dysfunctional uteri.

**Conclusions:**

The results indicate significant changes in expression of pluripotency markers in adenomyotic compared to normal uteri, which suggest the involvement of uterine stem cells in adenomyosis.

## Background

Adenomyosis is uterine dysfunction characterised by the presence of endometrial glands with stromal elements in the myometrium [1]. This pathological condition is well recognized in women, and although it is less known in domestic animals including cows [2–4], nevertheless it may result in reduced reproductive performance [4]. Although adenomyosis frequently occurs in multiparous women [5] and cows older than 5 years [4], the aetiology of this disorder is still unclear [2]. Several hypotheses have been proposed to explain adenomyosis development. One possible mechanism involves the breakdown of endometrial and myometrial barrier preceded by trauma such as abortion or gynaecological interventions, and followed by reactive hyperplasia of the endometrium and its proliferation within the myometrium [1, 6]. Another proposed mechanism of adenomyosis development involves metaplasia of uterine pluripotent/multipotent cells under hormonal stimuli. According to this hypothesis, glandular nests may arise *de novo* within the myometrial layer from undifferentiated stem cells under specific conditions, in particular under the influence of oestradiol (E_2_) [7, 8]. Whatever the mechanism underlying formation of glandular foci in the myometrium, hormonal and immunological abnormalities certainly play a role during adenomyosis development [9, 10].

Stem cells reside in many adult organs and tissues that exhibit high regenerative potential [11]. The cells may be identified by several markers, including NANOG, OCT4 and SOX2. These proteins are transcription factors present in embryonic stem cells [12] and, as recent studies have shown, in mesenchymal stem cells settled also in reproductive organs [13, 14]. OCT4 and SOX2 are progenitor-specific proteins: octamer-binding transcription factor 4 (OCT4) and sex determine region Ybox 2 (SOX2). NANOG is a homeodomain-containing transcription factor and its expression is regulated by OCT4/SOX2 heterodimer, which binds to the octamer/sox elements at NANOG gene promoter [15]. In the present study we selected NANOG, OCT4 and SOX2 as the markers of undifferentiated state and pluripotency/multipotency of cells that reside in uterus.

Changes that occur in the endometrium during reproductive cycles, in particular endometrial gland morphogenesis, require a remarkable proliferation capacity of the tissue; thus, pluripotent/multipotent cells play an important role in endometrial functioning and renewal [11, 16, 17]. These proliferative processes in the uterus remain under the strict control of ovarian steroids, therefore these hormones also influence uterine stem cell properties [11, 17].

During adenomyosis in cows, protein expression of the E_2_ receptor α (ERα) is increased [4], and also blood and endometrial E_2_ concentrations are elevated, which indicate hormonal abnormalities during this condition [4]. Parallel with increased E_2_ stimulation, excessive proliferation of endometrial cells occurs, which is characterized by expression of the proliferation marker KI-67-antigen in adenomyotic foci [18]. In our recent studies, we identified pluripotent/multipotent cells in the bovine uterus [19]. We also demonstrated expression of the pluripotency markers NANOG, OCT4 and SOX2 in uterine tissue and cultured uterine primary epithelial, stromal and myometrial cells, and in addition we confirmed pluripotent/multipotent properties of these cells by multilineage differentiation [19]. These results suggest that stem cells may be involved in adenomyosis development in the bovine uterus. Therefore, we hypothesized that pluripotency markers NANOG, OCT4 and SOX2 are differentially expressed in uterine tissues and cells from control and adenomyotic cows. The study by Moreira et al. (2007) showed increased frequency of adenomyosis in cows in the mid luteal stage of the oestrous cycle [20], so for this study we used uteri from cows at days 8–10 of the oestrous cycle.

The aims of the study were: (1) comparison of NANOG, OCT4 and SOX2 mRNA expression, immunolocalisation and protein expression in control and adenomyotic uterine tissues; (2) determination of NANOG, OCT4 and SOX2 mRNA and protein expression in cultured primary uterine endometrial stromal and myometrial cells isolated from adenomyotic cows.

## Methods

### Material collection

All procedures were approved by the Local Animal Care and Use Committee, Olsztyn, Poland (agreement no. 83/2012/N).

A total of 24 Holstein/Polish Black and White cows (75 %/ 25 %, respectively) 5–7 years old were used for *post mortem* collection of uteri (days 8–10 of the oestrous cycle). Uterine tissues were obtained at the Meat Processing Plant “Warmia” (Biskupiec, Poland) and transported on ice to the laboratory within 40 min. Day of the oestrous cycle was evaluated by macroscopic observation of the ovaries and uterus [21] and confirmed by determination of P_4_ levels in peripheral blood plasma using radioimmunoassay (RIA). Just before slaughter, each animal was examined by a veterinarian via *per rectum* ultrasound-guided examination and information about the age of each cow was recorded. Peripheral blood samples were collected from the jugular vein. The reasons for culling animals from the herd were economic considerations and herd renewal. For further experiments, after histopathologic examination, material quality evaluation and hormone determination, 18 cows were eventually selected (9 for each experimental group).

Tissue fragments (cross-sections of the uterine wall, i.e., endometrium and myometrium) were obtained from the middle segment of the uterine horn ipsilateral to the corpus luteum and were divided into three pieces: the first one was fixed in 4 % paraformaldehyde (PFA) in 0.1 M PBS (pH 7.4) for histo- and immunohistochemical staining, the second was frozen and stored at −86 °C for further mRNA and protein expression determination in whole uterine tissue, and the third piece was used for immediate isolation and culture of uterine cells.

### Histochemical staining and preliminary division of the material

**Fig. 1:**
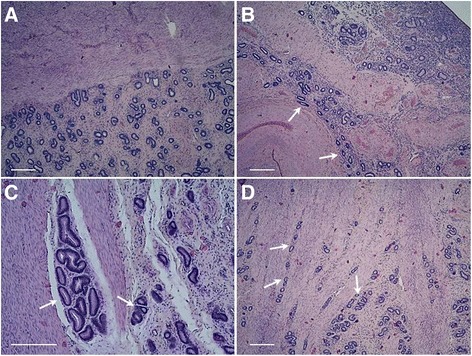
Representative pictures of haematoxylin and eosin stained bovine uterine cross-section slides. **a** – normal uterine tissue with regular, clearly visible border between endometrium and myometrium; **b**-**d** – adenomyotic tissue with visible uterine glands within the myometrial layer of the uterus. Arrows indicate glandular nests in myometrium. Scale bars: 100 μm

Uterine tissue was fixed in 4 % PFA and processed for a standard haematoxylin and eosin staining protocol. Stained cross-sections of the tissue were observed under a light microscope (Nikon FXA, Tokyo, Japan). Animals were classified as described previously [4]; briefly, if uterine glands were present only in the endometrium, and if the endometrial-myometrial border was clearly visible, cows were classified as normal/control (*n* = 9, Fig. 1a). Whereas, if the glands penetrated the myometrial layer of the uterus, animals were classified as adenomyotic (*n* = 9, Fig. 1b–d; according to the classification of Katkiewicz et al. 2005 [18]).

### Uterine cell isolation and *in vitro* culture

Endometrial stromal cells were isolated by enzymatic dissociation as previously described [22]. After endometrial cell isolation, the myometrial layer of the uterus was accessed and dissected with scissors. About 4 cm long fragments of muscle tissue were chopped up with scissors into a homogeneous material. Approximately 5 g of the chopped tissue was digested in 50 ml of M199 medium (Sigma, M2520, St. Louis, MO, USA) containing 0.1 % of bovine serum albumin (BSA; Sigma, A2058), 20 μg/ml of gentamicin (Sigma, G1271), 2 mg/ml of collagenase I (Sigma, C0130), 1 mg/ml of deoxyribonuclease (Sigma, D5025) and 2 mg/ml of dispase (Life Technologies, 17105–041, Paisley, UK). The enzyme solution with myometrial tissue was held at 37.5 °C with stirring for 30 min. After digestion, the cell suspension was filtered through a mesh to remove undigested tissue fragments, then the cells were washed by centrifugation (10 min at 100 x g, at 4 °C). Cells were resuspended in culture medium (DMEM; Sigma, D5796) supplemented with 10 % of fetal calf serum (FCS; Sigma, 12133C) and antibiotics (gentamicin/amphotericin B; Life Technologies, 1153727).

**Fig. 2:**
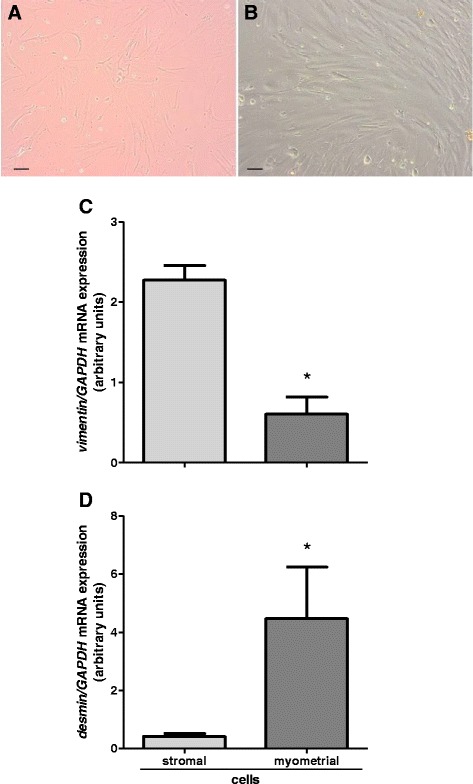
Evaluation of cell cultures homogeneity. **a**, **b** – representative pictures of stromal and myometrial cultured cells, respectively. Scale bars: 20 μm. Expression of vimentin in stromal and myometrial cells **c**; expression of desmin in stromal and myometrial cells **d**. Data were normalized against glyceraldehyde-3-phosphate dehydrogenase (*GAPDH*). Bars represent the mean ± SEM. Asterisks indicate statistical difference between uterine stromal and myometrial cells (*P* < 0.05), as determined by Student’s *t*-test

**Fig. 3:**
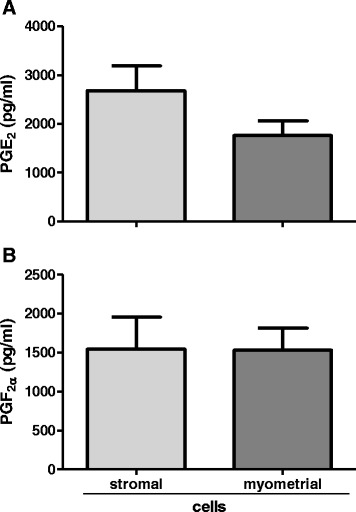
Prostaglandins secretion by stromal and myometrial cultured cells. Secretion of prostaglandin E_2_ by cultured uterine cells **a**; secretion of prostaglandin F_2α_ by cultured uterine cells **b**. Bars represent the mean ± SEM. There were no statistically significant differences in PGs secretion between stromal and myometrial cells (*P* > 0.05), as determined by Student’s *t*-test

The cells of each layer of the uterus were seeded separately at a density of 1 x 10^6^ living cells/ml in 1 ml and 2 ml culture medium per well in collagen-coated 24-well and 6-well plates, respectively (Biocoat; BD Bioscience, 4408, 4400, Bedford, MA, USA), and cultured at 37.5 °C in a humidified atmosphere of 5 % CO_2_, 95 % air. The medium was changed every 2 days until 70 % confluence was reached (approx. at 4^th^ day of culture). Total mRNA, cell lysates and culture media were collected. Stromal cells maintained during culture fibroblast-like morphology (Fig. 2a), while myometrial cells exhibited fusiform appearance (Fig. 2b). Purity of the cell cultures was rated by 4 independent observations under the light microscope based on cells morphology [23], and was evaluated for approx. 90–95 % for each cell types. Cell culture homogeneity was also confirmed using real-time PCR for determination of mRNA expression of vimentin and desmin for stromal and myometrial cells, respectively [23, 24]. Vimentin was highly expressed in stromal cells and in contrary weakly expressed in myometrial cell cultures (Fig. 2c). Desmin was expressed mainly in myometrial cultures, while in stromal cultures the expression was on low level (Fig. 2d). For cells’ functionality confirmation prostaglandin (PG) E_2_ and PGF_2α_ level was measured in culture medium by enzyme immunoassay (EIA; Fig. 3a, b). The levels of secreted PGs indicate maintained functionality of the uterine cells during cultures [25–27].

### Immunohistochemistry

Immunohistochemistry (IHC) was used to localise nuclear transcription factors, NANOG, OCT4 and SOX2, in uterine tissues.

Cross-sections of uterine horn samples were fixed in 4 % PFA in 0.1 M PBS (pH 7.4), and cryoprotected in 18 % sucrose. Immunostaining was carried out on consecutive 7 μm cryostat sections. To block endogenous peroxidase, the sections were treated with hydrogen peroxide in methanol and washed in 0.1 M PBS. The sections were blocked with 10 % normal goat serum (Sigma, G9023) for 1 h at room temperature (approx. 23 °C; RT), incubated overnight at RT with a 1:100 dilution of anti-NANOG (Abcam, 80892, Cambridge, UK), anti-OCT4 (Abcam, 19857) or anti-SOX2 (Sigma, S9072) antibodies, washed in PBS, incubated for 1 h with a 1:25,000 dilution of biotinylated anti-rabbit (Vectastain ABC Kit; Vector Laboratories, PK 4001, Burlingame, CA, USA) antibodies, then washed, incubated for 45 min with the ABD reagent in PBS, and washed again. Proteins were visualized by incubating the sections in 0.3 mg/mL 3,30-diaminobenzidine tetrahydrochloride in 0.01 % hydrogen peroxide in Tris-buffered saline (pH 7.2) for 2–3 min. Finally, the sections were dehydrated and cover-slipped with DPX mounting medium (Park Scientific Ltd, D-11601, Northampton, UK). To determine the specificity of the immunohistochemical staining, two controls were performed: first, the primary antibody was omitted during the immunostaining procedure; second, the primary antibody was substituted with a nonspecific IgG. Observations and photographs were made with a light microscope (Nikon FXA).

### Total RNA isolation

Total RNA was extracted from uterine tissues (approx. 30 mg) and cultured cells using TRI-Reagent (Sigma, T9424) according to the manufacturer’s instructions. The content and purity of RNA was assessed on a NanoDrop 1000 (Thermo Fisher Scientific, ND-1000, Wilmington, DE, USA). 260/280 absorbance ratio for all samples was approx. 2.0, and 260/230 absorbance ratio ranged between 1.8–2.2. One microgram of each sample of total RNA was reverse-transcribed to cDNA with the QuantiTect Reverse Transcription kit (Life Technologies, 205313), as described in the supplier’s protocol. The cDNA obtained was stored at −20 °C until real-time PCR was applied.

### Real-time PCR quantification

mRNA expression for *NANOG*, *OCT4* and *SOX2* in the tissues and cells was determined by quantitative real-time PCR. The experiments were performed using the Applied Biosystems 7900 (Applied Biosystems, Foster City, CA, USA) with SensiFAST SYBR Hi-ROX Kit (Bioline Reagents, BIO-92002, London, UK) according to the manufacturer’s instructions. The real-time PCR reaction mix (20 μl) contained 19 μl of SensiFAST SYBR Hi-ROX Master Mix, 0.5 μM of sense and antisense primers, and 1 μl of reverse-transcribed cDNA (50 ng). Primer sequences used for determination of vimentin, desmin, *NANOG*, *OCT4*, *SOX2* and glyceraldehyde 3-phosphate dehydrogenase (*GAPDH*) mRNA expression are detailed in Table 1. Standard curves consisting of serial dilutions of the appropriate cDNA were plotted for efficiency evaluation. Amplification was preceded by an initial enzyme activation step (2 min, 95 °C). The PCR steps were as follows: 40 cycles of denaturation (5 s, 95 °C), then annealing and extending (20 s, 60 °C). After amplification, melting curves were acquired by stepwise increases at a temperature of 50–95 °C to ensure that a single product was amplified and no primer-dimer structures were formed. Control reactions in the absence of the template or primers were performed to confirm that products were free from primer-dimers and genomic DNA contamination, respectively. Dissociation curves analysis was carried out after each real-time experiment to confirm the presence of only one amplification product. Data were normalized using the ΔΔC_t_ method. Samples were amplified in duplicates. Data are shown as the average fold increase, with S.E.M., and are expressed relative to the housekeeping gene *GAPDH*.

**Table 1:** Oligonucleotide sequences used for real-time PCR

Gene	Oligonucleotide sequences	Product size	GeneBank
		(bp)	
vimentin	FWD 5’-GACCTGGAGCGTAAAGTGG-3’	108	BC118269
	REV 5’-GACATGCTGTTCTTGAATCTGG-3		
desmin	FWD 5’-GACCCAGGCAGCCAACAAG-3’	100	BC133410
	REV 5’-GTCGATCTCGCAGGTGTAGG-3’		
NANOG	FWD 5’-TGCATTTGCTGGAGACTGAG-3’	107	DQ069776
	REV 5’- GTCCCGGTCAAGAAACAAAA-3’		
OCT4	FWD 5’-AGGTGTTCAGCCAAACGACTA-3’	195	FD381287.1
	REV 5’-TCTCCTGCAGATTCTCGTTGT-3’		
SOX2	FWD 5’-GCACATGAACGGCTGGAGCAACG-3’	218	JQ231229.1
	REV 5’-TGCTGCGAGTAGGACATGCTGTAGG-3’		
GAPDH	FWD 5’-CACCCTCAAGATTGTCAGCA-3’	103	BC102589
	REV 5’-GGTCATAAGTCCCTCCACGA-3’		

### Western blotting

Protein expression for NANOG, OCT4 and SOX2 in the tissues and cells was determined by Western blotting. Proteins from homogenised tissues and *in vitro* cultured cells were released by incubation with lysis buffer containing 50 mM Tris–HCl (pH 8.0), 150 mM NaCl, 5 mM EDTA, 0.1 % SDS, 1 % TritonX-100, 0.5 % sodium deoxycholate and protease inhibitors (Sigma, P8340). The lysates were stored at −86 °C until further analysis. Protein concentrations were measured by the Bradford’s method.

Western blot analysis was performed as previously described [28]. Equal amounts of protein were dissolved in SDS gel-loading buffer, heated to 95 °C for 4 min and separated in 10 % SDS-PAGE. Separated proteins were electroblotted onto 0.2 μm nitrocellulose membranes in transfer buffer. After blocking in 5 % non-fat dry milk in TBS-T buffer for 1.5 h at RT, the membranes were incubated overnight with a 1:250 dilution of anti-NANOG (Novus Biologicals Ltd, NBP2-24941, Cambridge, UK), a 1:400 dilution of anti-OCT4 (Novus Biologicals Ltd, NB100-2379) or a 1:500 dilution of anti-SOX2 (Sigma, S9072) antibodies; GAPDH (Sigma, G8795; monoclonal anti-glyceraldehyde-3-phosphate dehydrogenase antibody produced in mouse) expression was used as a reference. Proteins were detected by incubating the membranes with a 1:20,000 dilution of secondary polyclonal anti-rabbit or anti-mouse alkaline phosphatase-conjugated antibodies (Sigma, A 3687, A 3562) for 1.5 h at RT. Western blots were quantitated using the Kodak 1 D program (Eastman Kodak, Rochester, NY, USA).

### Hormone determination

Measurements of P_4_ in blood plasma were performed using a direct radioimmunoassay (RIA; DIASource ImmunoAssays S.A., KIP1458, Nivelles, Belgium). The standard curve ranged from 0.12–36 ng/ml and the effective dose for 50 % inhibition (ED 50) of the assay was 0.05 ng/ml. The intra- and inter-assay coefficients of variation (CV) were 6.5and 8.6 %, respectively.

### Prostaglandins determination

Measurements of PGE_2_ and PGF_2α_ levels in culture media were performed using commercially available enzyme immunoassay kit (EIA kit; Cayman Chemical Company, 514010 for PGE_2_ and 516011 for PGF_2α_, Ann Arbor, MI, USA). Standard curve for PGE_2_ ranged from 9,5–5000 pg/ml and the effective dose for 50 % inhibition (ED 50) of the assay was 15 pg/ml. The intra- and inter-assay coefficients of variation (CV) were 4.2 % and 12.4 %, respectively. PGF_2α_ standard curve ranged from 9,5–2000 pg/ml, ED 50 of the assay was 9 pg/ml and the intra- and inter-assay CV were on average 9.4 % and 12 %, respectively.

### Statistical analysis

Statistically significant differences between groups in the experiments were evaluated using Student’s *t*-test (GraphPad PRISM Version 5.00, San Diego, CA, USA). All data were expressed as means ± SEM. Differences were analysed between control and adenomyotic cows, and were considered significant at *P* < 0.05.

## Results

### mRNA expression, immunolocalisation and protein expression of pluripotency markers NANOG, OCT4 and SOX2 in uterine tissue of cows with adenomyosis

**Fig. 4:**
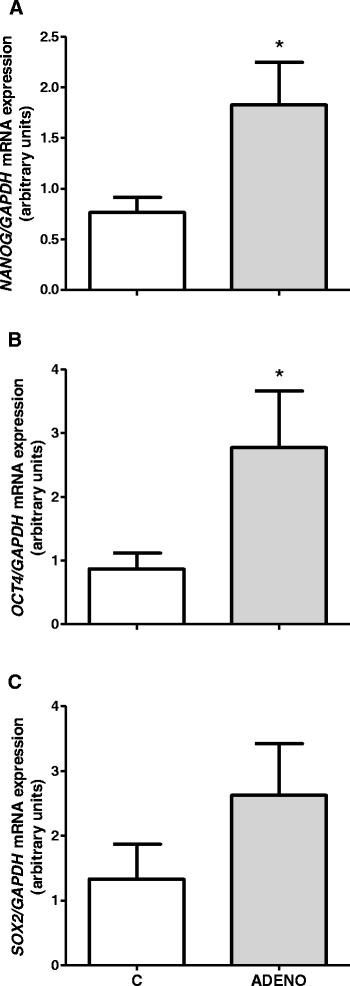
mRNA expression of pluripotency markers *NANOG* **a**, *OCT4* **b** and *SOX2* **c** in uterine tissues obtained from control cows and from cows with adenomyosis. Data were normalized against glyceraldehyde-3-phosphate dehydrogenase (*GAPDH*). Bars represent the mean ± SEM. Asterisks indicate statistical difference between uterine normal and adenomyotic tissues (*P* < 0.05), as determined by Student’s *t*-test. C – tissues obtained from control cows, ADENO – tissues obtained from cows with adenomyosis

mRNA expression of transcription factors *NANOG* and *OCT4* was increased in adenomyotic uterine tissue compared with normal uteri (*P* < 0.05, Fig. 4a, b). There was no significant difference in *SOX2* mRNA expression between control and adenomyotic uteri (*P* > 0.05, Fig. 4c).

**Fig. 5:**
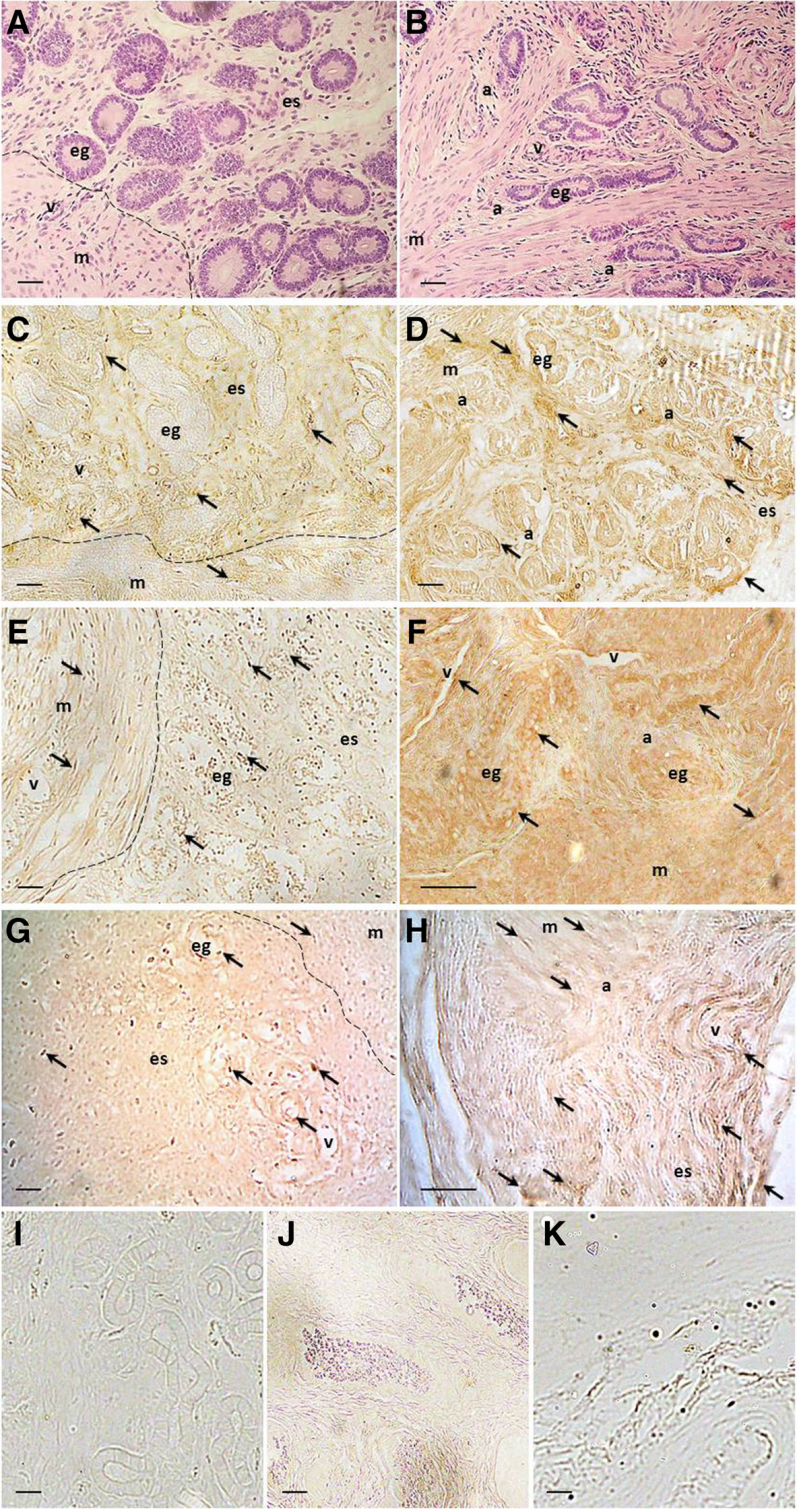
Immunodetection of pluripotency markers in uterine tissues from control cows and from cows with adenomyosis. **a**, **b** – haematoxylin and eosin stained slides of control and adenomyotic uterus, respectively; **c**, **d** – NANOG immunodetection in control and adenomyotic tissue, respectively; **e**, **f** – immunolocalisation of OCT4 in normal and adenomyotic tissue, respectively; **g**, **h** – SOX2 immunodetection in control and adenomyotic tissue, respectively; **i**, **j**, **k** – no Ab, negative controls for NANOG, OCT4 and SOX2, respectively. Unspecific IgG controls (pictures not shown) served similar pictures as no Ab control. Arrows indicate the most intense histochemical reactions; dotted line indicate endometrial-myometrial border; e – endometrium, m – myometrium, es – endometrial stroma, eg – endometrial gland, a – adenomyotic lesion, v – vessel. Scale bars: 20 μm

Immunohistochemistry revealed expression of all three pluripotency markers, NANOG, OCT4 and SOX2, in normal uterine tissues (Fig. 5c, e, g) as well as in adenomyotic samples (Fig. 5d, f, h). The proteins examined were mainly localised in the endometrium, however, in the case of adenomyotic tissues the immunoreactivity was also high in the myometrial compartment of the uterus and in adenomyotic, ectopic glands (Fig. 5d, f, h).

**Fig. 6:**
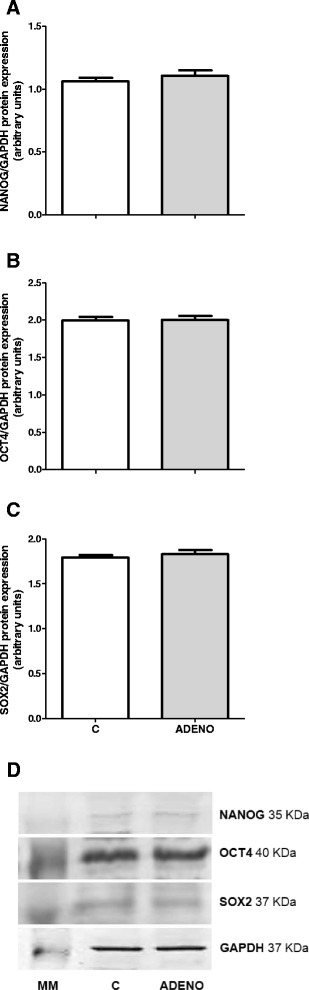
Protein expression of NANOG **a**, OCT4 **b** and SOX2 **c** in bovine uterine tissues obtained from control cows and from cows with adenomyosis. Data were normalized against glyceraldehyde-3-phosphate dehydrogenase (*GAPDH*). Bars represent the mean ± SEM. There were no statistical differences between uterine normal and adenomyotic tissues (*P* > 0.05), as determined by Student’s *t*-test. Representative blots for NANOG, OCT4, SOX2 and GAPDH are shown below the graphs **d**. MM – molecular weight marker, C – tissues obtained from control cows, ADENO – tissues obtained from cows with adenomyosis

At the protein expression level, determined by Western blotting, there were no significant differences among NANOG, OCT4 and SOX2 (*P* > 0.05, Fig. 6a–c). However the spatial differences in examined proteins expression was reported during IHC assay.

### mRNA and protein expression of pluripotency markers NANOG, OCT4 and SOX2 in uterine cells from cows with adenomyosis

**Fig. 7:**
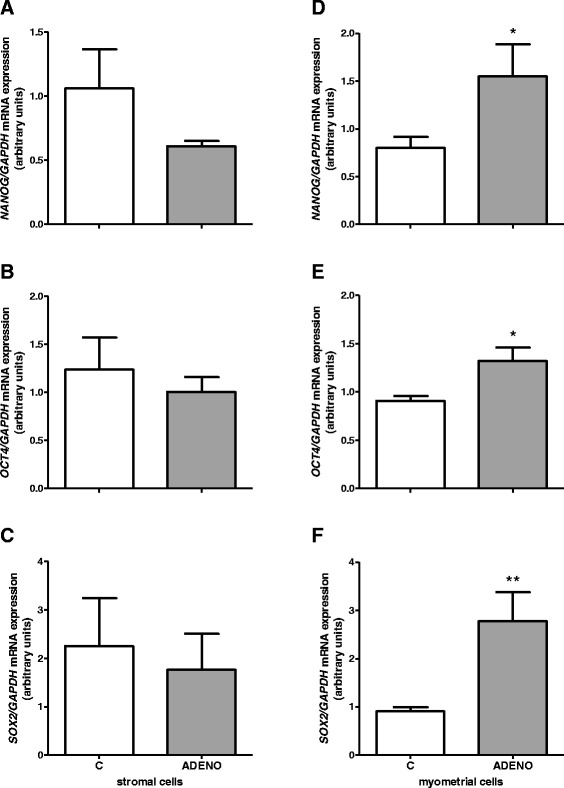
mRNA expression of pluripotency markers in uterine cells isolated from control cows and from cows with adenomyosis. *NANOG* **a** *OCT4* **b** and *SOX2* **c** mRNA expression in uterine stromal cells. *NANOG* **d** *OCT4* **e** and *SOX2* **f** mRNA expression in uterine myometrial cells. Data were normalized against glyceraldehyde-3-phosphate dehydrogenase (*GAPDH*). Bars represent the mean ± SEM. Asterisks indicate statistical differences between uterine normal and adenomyotic tissue (*P < 0.05; **P < 0.01), as determined by Student’s *t*-test. C – cells obtained from control cows, ADENO – cells obtained from cows with adenomyosis

Expression of all genes of the three transcription factors did not differ significantly between cultured stromal cells isolated from adenomyotic uteri compared to those isolated from control tissues (*P* > 0.05, Fig. 7a–c). Whereas, mRNA expression of *NANOG*, *OCT4* and *SOX2* in cultured uterine myometrial cells isolated from cows with adenomyosis was increased compared to those isolated from normal uteri (*P* < 0.05, Fig. 7d–f).

**Fig. 8:**
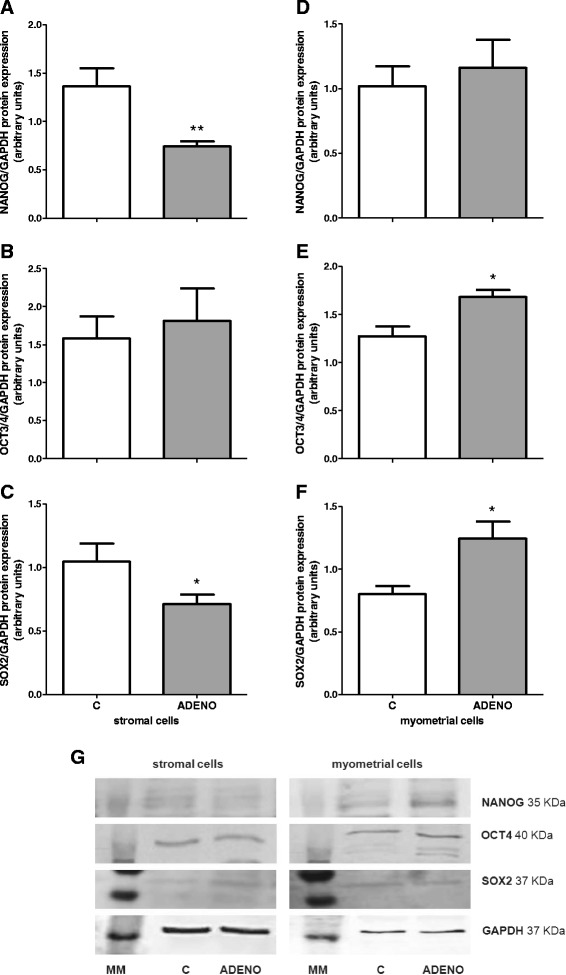
Protein expression of pluripotency markers in uterine cells isolated from control cows and from cows with adenomyosis. NANOG **a** OCT4 **b** and SOX2 **c** protein expression in uterine stromal cells. NANOG **d** OCT4 **e** and SOX2 **f** protein expression in uterine myometrial cells. Data were normalized against glyceraldehyde-3-phosphate dehydrogenase (*GAPDH*). Bars represent the mean ± SEM. Asterisks indicate statistical differences between uterine normal and adenomyotic tissue (*P < 0.05; **P < 0.01), as determined by Student’s *t*-test. Representative blots for NANOG, OCT4, SOX2 and GAPDH are shown below the graphs **g**. MM – molecular weight marker, C – cells obtained from control cows, ADENO – cells obtained from cows with adenomyosis

Protein expression of NANOG and SOX2 was significantly decreased in stromal cells isolated from uteri with adenomyosis compared to those obtained from normal uteri (*P* < 0.05, Fig. 8a, c). Protein expression of both transcription factors OCT4 and SOX2 was higher in cultured myometrial cells from adenomyotic tissues than in corresponding cells isolated from normal uteri (*P* < 0.05, Fig. 8e, f).

## Discussion

Adenomyosis is a uterine proliferative dysfunction which aetiology is still unclear. One possible mechanism of its development implies the involvement of uterine stem cells, which abnormal proliferation and differentiation may lead to formation of glandular foci within the myometrium [2, 12]. In our previous study, we confirmed the existence of pluripotent/multipotent cells in the bovine uterus [19]. The present research focused on determining expression of pluripotency markers in adenomyotic uterine tissues and cells. We confirmed that pluripotency markers are expressed in adenomyotic endometrial tissues and in glandular nests within the myometrium, and that mRNA expression for NANOG and OCT4 was higher in dysfunctional tissue compared to the control; however, the tissue protein results determined by Western blotting did not confirm these differences. Moreover, we have demonstrated that both mRNA and protein levels for OCT4 and SOX2 were increased in cultured primary myometrial cells isolated from adenomyotic uteri compared to the cells isolated from normal tissues. However, in stromal cells protein expression of NANOG and SOX2 was significantly decreased in the case of adenomyosis. To our knowledge, this is the first study to report expression of pluripotency markers in the bovine uterus with adenomyosis.

In women, the hypothesis of uterine stem cell involvement in development of uterine dysfunction has been widely studied [11, 29–31]. Moreover, the recent report of Chen et al. (2014) showed increased expression of another stem cell marker, Musashi-1, in adenomyotic eutopic and ectopic endometrium of women [32]. The presence of pluripotent/multipotent cells in the bovine uterus suggests their possible role in development of uterine dysfunction [19, 33, 34]. Nevertheless, there are no studies on the involvement of pluripotent cells in pathogenesis of adenomyosis or any other uterine diseases in cows. Our present study is consistent with one conducted in women [32], because we demonstrated increased expression of pluripotency markers in myometrial cells isolated from adenomyotic uteri. However, in whole uterine tissue, at the protein level evaluated by Western blotting we did not find significant differences in NANOG, OCT4 and SOX2 expression, in contrast to our mRNA results. Moreover, mRNA expression of pluripotency markers in stromal primary cells also did not reflect protein expression. The reason for this inconsistency may be posttranslational modifications of the proteins, which results in different protein expression pattern when comparing to mRNA. However in myometrial cells mRNA expression was consistent with protein expression. These outcomes together with IHC results indicate that the pattern of pluripotency markers expression in adenomyotic tissue may depends on the uterine compartment: endometrium or myometrium. Our experiments performed on the tissue showed general expression of pluripotency markers in adenomyotic uterus, which included its expression in stem cells that migrate to uterus through blood and lymphatic vessels, e.g. cells of medullary origin [31]. Whereas *in vitro* experiment revealed pluripotency markers expression in particular uterine cells, originated from endometrium or myometrium. In our previous study we showed that the main source of stem cells in the bovine uterus is stromal layer [19], therefore we showed changed expression pattern of pluripotency markers in case of uterine pathology – adenomyosis.

In our study, we also demonstrated decreased protein expression of NANOG and SOX2 in cultured primary endometrial stromal cells from adenomyotic tissues compared to the controls, which suggests that their potential to differentiate into glands within the endometrium may be reduced. Proliferation of endometrial cells and formation of uterine glands is extremely important for successful implantation and early embryo development [35]. In high fertility heifers, endometrial expression of genes involved in cell proliferation, tissue morphology and development was increased when compared to low fertility heifers [36]. Thus, during adenomyosis, disturbed proliferative processes in the endometrium of cows may impair their fertility [18, 37]. Moreover, because the myometrium is a prolific source of pluripotent cells during adenomyosis, this may imply a higher differentiation potential of cells in this compartment of the uterus, and may trigger the invasion of glandular nests deep within.

Maintenance of uterine cell functions, including proliferation, during cyclic endometrial remodelling is the main factor underlying female fertility in many species and is controlled by ovarian hormones [29, 37–39]. In women, abnormalities in endometrial cell proliferation potentially leads to development of gynaecological diseases such as endometriosis, endometrial cancers and adenomyosis [11, 29]. Bovine endometrial cell proliferation is also regulated by ovarian hormones: oestradiol and progesterone [40]. Adenomyosis is an oestrogen-dependent dysfunction [4, 30], thus it is suggested that hormone disturbances may relate to uterine stem cell functioning during adenomyosis [29]; however, this issue require further studies. In mares, defective responses of endometrial glands to cyclic hormonal stimuli may contribute to degenerative changes in the endometrium, termed endometrosis, and result in decreased fertility [41]. These changes are also linked to functional abnormalities of endometrial cells, especially impaired proliferation activity in endometrotic glandular nests [39]. Although knowledge about the involvement of uterine stem cells in pathogenesis of endometrosis is poor, recent studies on mare infertility caused by this dysfunction suggest the utility of stem cell transplantation into uteri for therapy. Stem cells that settled in degenerative endometria during the experiments of Mambelli et al. (2014) induced proliferation of glands and improved their secretory functions [42]. These data suggest that a wide range of uterine pathologies in different species may be dependent on functions of uterine stem cells. Therefore, our present study contributes by broadening knowledge about this issue in cattle, which was not previously studied in this species.

## Conclusions

In conclusion, our results indicate that significant changes occur in the expression of pluripotency markers, NANOG, OCT4 and SOX2, in bovine adenomyotic tissues compared to normal uteri. Moreover, this expression was dependent on the uterine compartment: in general, it was decreased in the endometrial layer and the converse in the myometrial layer. These data suggest the involvement of uterine multipotent/pluripotent cells in development of adenomyosis. Therefore our study imply that stem cells play role not only under physiological conditions but also in the case of uterine pathologies in cows.
